# Prep provision in community organizations: a comparative study with conventional services

**DOI:** 10.11606/s1518-8787.2024058005914

**Published:** 2024-10-11

**Authors:** Alexandre Grangeiro, Paula Andrea Massa, Maria Mercedes Escuder, Eliana Miura Zucchi, Eliane Aparecida Sala, Eduardo Araujo de Oliveira, Raphaela Fini, Inês Dourado, Laio Magno, Beo Oliveira Leite, Katia Bruxvoort, Sarah MacCarthy, Marcia Thereza Couto, Maria Fernanda Tourinho Peres

**Affiliations:** I Universidade de São Paulo. Faculdade de Medicina. São Paulo, SP, Brasil Universidade de São Paulo Faculdade de Medicina SP São Paulo Brasil; II Secretaria de Estado da Saúde de São Paulo. Instituto de Saúde. São Paulo, SP, Brasil Secretaria de Estado da Saúde de São Paulo Instituto de Saúde SP São Paulo Brasil; III Universidade Católica de Santos. Programa de Pós-Graduação em Saúde Coletiva. Santos, SP, Brasil Universidade Católica de Santos Programa de Pós-Graduação em Saúde Coletiva SP Santos Brasil; IV Universidade Federal da Bahia. Instituto de Saúde Coletiva. Salvador, BA, Brasil Universidade Federal da Bahia Instituto de Saúde Coletiva BA Salvador Brasil; V Universidade do Estado da Bahia. Departamento de Ciências da Vida. Salvador, BA, Brasil Universidade do Estado da Bahia Departamento de Ciências da Vida BA Salvador Brasil; VI University of Alabama at Birmingham. Birmingham, AL, United States of America University of Alabama at Birmingham AL Birmingham United States of America

**Keywords:** HIV, Pre-Exposure Prophylaxis, Communitarian Organization, Adolescent, Intervention Study

## Abstract

**OBJECTIVE:**

To evaluate whether adolescents from sexual minorities who initiated pre-exposure prophylaxis (PrEP) in community-based organizations (COs) are more socially and HIV-vulnerable compared with their counterparts from a conventional health service. In addition, to evaluate whether these adolescents had more timely access to prophylaxis

**METHODS:**

A PrEP demonstration study was conducted in the city of São Paulo in two COs, located in the center (CO-center) and the outskirts (CO-outskirts), and a conventional HIV testing service (CTA-center). Between 2020 and 2022, cisgender male adolescents who have sex with men (aMSM), transgender and gender diverse adolescents (aTTrans) aged 15 to 19 years, HIV-negative, with higher-risk practices for HIV were eligible for PrEP. Indicators of timely access and vulnerabilities of adolescents initiating PrEP in COs were analyzed using CTA-center as a reference and multinomial logistic regression.

**RESULTS:**

608 adolescents initiated PrEP in COs and CTA-center. Adolescents from COs were associated with a shorter time to PrEP initiation (1–7 days; CO-outskirts: ORa = 2.91; 95%CI 1.22–6.92; CO-center: ORa = 1.91; 95%CI 1.10–3.31); and a lower housing Human Development Index (HDI) (CO-center: ORa = 0.97; 95%CI 0.94–1.00; CO-outskirts: ORa = 0.82; 95%CI 0.78–0.86). In CO-outskirts, there was an increased chance of adolescents being younger (ORa = 3.06; 95%CI 1.63–5.75) and living closer to the service (ORa = 0.82; 95%CI 0.78–0.86, mean 7.8 km). While adolescents from the CO-center were associated with greater prior knowledge of PrEP (ORa = 2.01; 95%CI 1.10–3.91) and high-risk perception (ORa = 2.02; 95%CI 1.18–3.44), adolescents from the COs were not associated with higher-risk sexual practices and situations of vulnerability to HIV.

**CONCLUSION:**

The provision of PrEP in the COs facilitated access for vulnerable adolescents and may contribute to reducing inequities.

## INTRODUCTION

Expanding the use of pre-exposure prophylaxis (PrEP) for people who are more socially and economically vulnerable to HIV is a challenge to achieving the goal of eliminating the HIV epidemic by 2030 ^1^ . This has led to several initiatives to simplify the provision of the prophylaxis, such as prescription by different professionals, provision outside of health services, and using communication technologies ^2^^-^^4^ . Despite this, most PrEP provisions focus on physicians and specialized services in high-, low-, and middle-income countries ^3^^,^^4^ . Significant barriers to access to services ^5^^,^^6^ also persist, such as stigma, fear of revealing sexual orientation and gender identity in unfriendly services, and the inadequacy between PrEP provision and people’s daily lives. In addition, adolescents have limited access to PrEP, leading to a limited presence of this population in services and studies on efficacy and effectiveness ^7^ . Therefore, producing more accurate knowledge appropriate to this population’s needs is required.

Many studies have evaluated alternatives for providing PrEP, recognizing that no single method covers the diversity of contexts and priority populations ^4^^,^^5^ . In this sense, providing PrEP within the community has proven effective, with experiences exploring different types of services and professionals responsible for prescribing. In Thailand and Vietnam ^8^^,^^9^ , community prescribing for cisgender men who have sex with men (MSM) and transgender women was performed by trained peers identified in sexual networks, improving the degree of identification of people at risk and the initiation rate of prophylaxis. In Namibia ^10^ , medical and nursing professionals prescribe for adolescents and young women in the community. The users choose the place and time to receive the prescription, resulting in improved use continuity compared with conventional health services.

Different professionals’ prescriptions have also been associated with increased access and improvements in the PrEP cascade. Nursing has gained prominence due to user acceptance ^11^ , motivation for prescribing ^12^^,^^13^ , and inclusion in activities that favor screening and follow-up of prophylaxis ^4^ . The increased role of nursing has been accompanied by the reformulation of PrEP protocols for the category’s work ^3^^,^^14^ and questions about the permanence of positive results from nursing work in non-specialized services ^4^ .

In Brazil, different professionals can prescribe PrEP in specialized services, primary care, and private practices using the national guideline ^15^ and the Unified Health System (SUS) medications. Despite this prophylaxis coverage remains low and concentrated in adults with higher socioeconomic status ^16^ . Therefore, in 2020, within the scope of the PrEP1519 study, a PrEP demonstration study was implemented in São Paulo to evaluate, among adolescents, the access and effectiveness of offering PrEP in two community organizations (COs). This article assessed whether adolescent cisgender men who have sex with men (aMSM), *travestis* (term traditionally used by the community in Brazil to characterize gender identity and political activism), transgender women, and transfeminine people (aTTrans), who used PrEP in these two COs, had more significant social and HIV vulnerability; and whether they had more timely access to prophylaxis when compared with adolescent users of a conventional health service.

## METHODS

### The PrEP1519 Study

This is a demonstration study of daily oral PrEP ^17^ , developed in Belo Horizonte, Salvador, and São Paulo, with adolescents aged 15 to 19 years who identified themselves as AMSM and aTTrans. PrEP was offered following the Brazilian protocol ^15^ , which defines as main prescription criteria having a negative anti-HIV test and having a higher risk or vulnerability to HIV and, as exclusion criteria, compromised renal function or not having sufficient understanding for adherence and permanence in PrEP. In São Paulo, the study began in February 2019 in one of the city’s most traditional testing and counseling centers in the central region (CTA-center), Sé neighborhood, with prescription by a medical team and counselor support. The present study defined CTA-center as a “conventional health service.” In 2020, the study was expanded, exclusively in the city of São Paulo, to offer PrEP in two COs with outreach teams. The objective was to compare this provision in the community with the conventional health service, assuming that providing prophylaxis in a more friendly and community-friendly context would reduce barriers to access for adolescents who are not regularly included in health services.

### Community Organizations

We define community organizations as those that result from citizens’ collective action in favor of a population’s interests and improvements. Thus, to allow for a better comparison, we selected a CO located in a region similar to that of CTA-center and another in an area substantially opposite concerning the concentration of wealth, population characteristics, and conditions of access to health. For this selection, formative research ^18^ was carried out in the eastern and central regions of São Paulo. In the center, in the Bela Vista neighborhood, an area adjacent to CTA-center, a CO (CO-center) was identified that works in social support and defense of LGBTQIAPN+ rights; and, in the far east, the Cidade Tiradentes neighborhood, an organization (CO-outskirts) that develops actions for the general population, aiming to respond to the challenges that affect the outskirts.

The three neighborhoods that house the organizations/services in the study differ ^19^ , among other aspects, in the proportions of Black people (21.6% – Bela Vista; 38.3% – Sé; 56.1% – Cidade Tiradentes); aged between 0 and 29 years (27.2% – Bela Vista; 39.2% – Sé; 48.5% – Cidade Tiradentes); who live within a 1 km radius of train/metro stations (86.4% – Sé; 54.7% – Bela Vista; 0.0% – Cidade Tiradentes); and in the average monthly income from work (R$ 4,403.34 - Bela Vista; R$ 4,197.02 – Sé; R$ 2,654.42 – Cidade Tiradentes).

### Protocol for Offering PrEP in Community Organizations

The protocol is detailed in the Chart 1 , as are the differences concerning the conventional service. In summary, each CO received an outreach team of nurses and peer educators selected from the community. This team was responsible for all activities related to demand creation, clinical care, linkage, adherence, and retention. A group of professionals from medicine, psychology, and social work was formed as a support team to support the discussion of cases and the monitoring of adolescents who required more complex or interdisciplinary approaches.

Demand creation and linkage strategies were developed to identify adolescents at greater risk for HIV in social environments, namely: “ *in the community* ,” involving meeting places, social networks, and date apps, and “ *within the institutions* ,” with the identification of adolescents in the clientele and/or referred by other institutions. These strategies, developed mainly by peer educators, also covered the COs and CTA-center ^20^ . In addition, CO-outskirts used an approach used in research with hard-to-reach populations called respondent-driven sampling (RDS) ^21^ . This strategy encourages a participant to invite three other potential participants, triggering successive “waves.”

Adolescents identified in demand creation who expressed interest in PrEP were then involved in the linkage strategies. In these, the adolescent was accompanied by a peer educator, virtually or in person, until they arrived at the organization.

Adolescents who arrived at the organizations were screened, started PrEP, and clinically followed according to the Ministry of Health protocol, which was simplified and detailed for application in a community context and prescription by nursing ( Chart 1 ). For sexually transmitted infections (STIs), the syndromic and etiological approach was used, with diagnosis and prescription of treatment performed at the institution and provision/administration of medications at a public health service.

**Chart 1.** Protocol for providing PrEP in community organizations. PrEP 1519 Study.

**Process**	**Community offering**	**Difference compared with conventional service** ^a^
**Team**	Outreach team working in each community organization: a nursing professional responsible for prescribing, managing STIs, adverse events, and counseling; and two peer educators responsible for creating demand, welcoming, linkage, retention, and discussion groups. Support team for the COs: medical, social work, and psychology professionals in charge of case discussion and follow-up of more complex cases.	Reception, nursing, nursing technician, counselors, and medicine professionals
**Training**	Continuing education, with monthly meetings, on topics related to sexuality, LGBTQUIAPN+, and prevention. Dynamics: discussion with external guests and case discussion.	Not performed
**Physical space**	Shared, consisting of clinical office, with collection area and counseling room	Exclusive use space
**Opening hours**	Weekdays: afternoon and evening; Saturdays: afternoon	Working days: morning and afternoon
**Demand creation**	Actions performed mainly by a peer educator who seeks to identify people who can benefit from PrEP, explain the benefits, and accompany them to the service when necessary. These actions were carried out in in-person socializing and party locations, virtual spaces on partner meeting apps, and other social networks like Instagram. It was called spontaneous demand when a participant recommended the service to others or when a service, institution, or healthcare professional recommended it. We also use the respondent-driven sampling (RDS) strategy in the CO-outskirts. In this study, male adolescents who have sex with men were encouraged to invite three other adolescents to respond to a behavioral survey and so on, forming a “kind of wave.” The incentive for the invitation was provided through financial assistance and a prevention kit. Adolescents eligible for PrEP, after responding to the behavioral survey, were invited to start PrEP after counseling.	Similar, except RDS
**Linkage**	Access/first consultation: Monitoring via virtual means and in person during travel (navigation), if necessary. Follow-up consultations: At least two virtual contacts in the first 30 days and one between consultations. For all consultations: Financial support for transportation and food is available. Responsible professional: peer educator.	Similar, performed by a healthcare professional
**Rapid testing**	Quarterly: HIV and Syphilis; semi-annual hepatitis C and B (without immunization)	Similar
**Chlamydia and gonorrhea**	Semi-annual: anal, urethral, oral (material obtained by self-collection)	Not available in the public network
**Collection of safety exams**	Annual: Kidney and liver function	Similar
**Management of adverse events**	Grade 1 and 2 events: care provided by nursing; grade 2 event: no remission of symptoms assessed by a medical professional; and grades 3 and 4: referral to emergency services.	Event with grade 1 attended by nurses/medical professionals and the rest by medical professionals
**Changes in laboratory exams**	Management defined according to DAIDS criteria, with initial nursing care. Referred for medical care: grade 2 events with no improvement; use of medications for chronic diseases and/or comorbidities; and asymptomatic grade 3/4 events.	Event with grade 1 attended by nurses/medical professionals and the rest by medical professionals
**Management of STI**	Syndromic and etiological diagnosis and prescription of treatment, such as provision of medication at the public health system.	Syndromic and etiological diagnosis and treatment
**Adherence and retention**	Care navigation with the provision of telecare, discussion groups, virtual or in-person support for access to the service and other services, psychosocial support provided by a peer educator and support team for more complex cases (social worker, psychologist), and Referral to the psychosocial care network, if required.	Psychosocial reception, referral to health care network
**Groups and discussion circles**	Group for gender affirmation and social inclusion of *travestis* and transgender women, Group for adolescent men who have sex with men, and Groups for adherence and linkage. Characteristics: open to adolescents on PrEP and the community every two weeks, mediated by a peer educator and professional support team.	Not performed

CO: community organization; STI: sexually transmitted infection; PrEP: pre-exposure prophylaxis; DAIDS: Division of AIDS Table for Grading the Severity of Adult and Pediatric Adverse Events; PCDT: Clinical Protocol and Therapeutic Guidelines; CTA: testing and counseling center.

a Based on the CTA-center-adopted protocol of the Municipal Health Department and the Clinical Protocol and Therapeutic Guidelines of the Ministry of Health.

**Figure 1.**
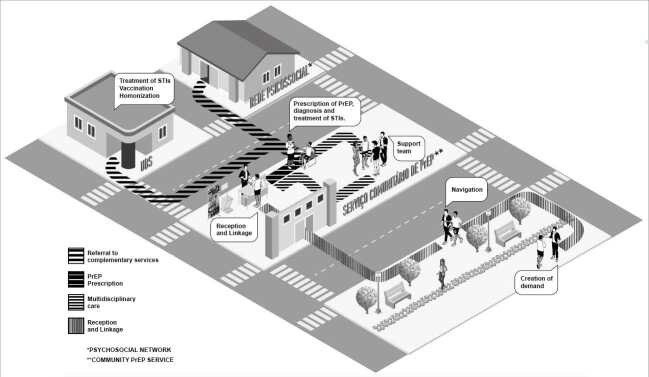
Components and flow of care in the provision of PrEP in community organizations. PrEP 1519 Study.

After the initial PrEP prescription, peer educators and support staff contacted the adolescent through virtual channels to support adherence, retention, or other psychosocial needs. PrEP support also involved reception, discussion groups, and referral to services. For aTTrans, a specific group was created to support the care process, addressing topics such as transphobia, gender affirmation, and social support. Figure 1 shows the flow of care.

### Population and Study Period

This study included adolescents who received at least one PrEP prescription in any of the three study services between February 2020 and February 2022.

### Assumptions and Variables

The selection of variables assumed that provision in a community setting, compared with the conventional service (CTA-center), would increase the chance of starting PrEP and that adolescents would have the following characteristics:

1. Timely access: Living closer to the organization and receiving the initial PrEP prescription within seven days after the first contact with peer educators. Distance from the place of residence was considered an indicator of timely access based on the assumption that provision in the community would reduce stigma and, thus, provide priority access for people living locally and in the shortest possible time.
2. Greater social vulnerability: We included adolescents from regions with a lower Human Development Index (HDI). We adopted the HDI because it better describes social vulnerability than individual characteristics. Adolescents, who comprise a narrow age range, usually have slight variations in education level, while they may know little about family income and/or housing situation.
3. Greater vulnerability to HIV: Lower level of information (not previously aware of PrEP and self-testing); low self-perception of risk (self-assessment of risk ≤ 6, on a scale of 10, and not using a condom during the first sexual intercourse); less access to prevention (never having taken post-exposure prophylaxis [PEP] or HIV testing); adoption of higher-risk practices (having a larger number of casual partners, anal sex without a condom and history of STIs); and being in contexts of vulnerability for prevention (sex work, use of marijuana and other drugs during sex).

The distance between home and institution was estimated using the Open Source Geographic Information System, version 3.32. The time of PrEP initiation was determined by the dates of first contact with a demand creation strategy and the initial PrEP prescription, categorized as: “same day,” “1 to 7 days,” and “≥ 8 days.” In São Paulo, we used the place of residence by Human Development Units (UDHs) to categorize HDI. UDH aggregates homogeneous and contiguous areas defined with data from census tracts ^22^ . For other cities, the reference was the municipal HDI.

The information was obtained from electronic care records in the study’s online system, implemented in the three institutions. Practices, perception, and context of vulnerability were related to the three months before inclusion and the others for the entire life.

### Statistical Analysis

Multinomial logistic regression with 95% confidence intervals (95%CI) was used to analyze the association of the variables of timely onset, social vulnerability, and vulnerability to HIV with COs, using CTA-center as a reference. All variables ( Tables 1 and 2 ) were included in the analysis, except for education, a parameter included in the HDI, and demand creation strategies, as they are intrinsic to the work process of organizations/services. Variables with a significance level of ≤ 0.1 in the bivariate analysis were included in the initial multivariate model, while those with a significance level of ≤ 0.05% remained in the final model. Missing data were excluded. Analyses were performed using SPSS, version 23.

Participants signed the Informed Consent Form. A court order exempted adolescents between 15 and 17 years old from parental consent (SP: 1128747-15.2018.8.26.0100). The protocol was approved by the Clinics Hospital Ethics Committee of the School of Medicine of the University of São Paulo, with opinion number 4,488,944.

## RESULTS

Six hundred-eight adolescents initiated PrEP, mostly in CTA-center (n = 409, 67.3%). Their sociodemographic profile differed (p < 0.05), exclusively due to the predominance of younger individuals (15–17 years, 42%), lower education levels (elementary school, 37.5%) in the CO-outskirts, and a higher proportion (30.8%) of adolescents with private health insurance in CTA-center ( Table 1 ).

**Table 1.** Characteristics of adolescent men who have sex with men, *travestis* , transgender women, and transfeminine people who initiated PrEP, in São Paulo, from February 2019 to February 2022. PrEP 1519 Study.

**Characteristics**	**CTA-center**		**CO-center**		**CO-outskirts**		**Total**		**p**
	n	%	n	**%**	n	%	n	%	
Total	409	67.3	111	18.3	88	14.5	608	100	
Demographics									
Gender									
Cisgender man	378	92.4	104	93.7	77	87.5	559	91.9	0.231
Transgender woman and *travesti*	31	7.6	7	6.3	11	12.5	49	8.1	
Age group									
15 to 17	92	22.5	33	29.7	37	42.0	162	26.6	< 0.001
18 to 19	317	77.5	78	70.3	51	58.0	446	73.4	
Skin color									
Black	234	57.2	68	61.3	56	63.6	358	58.9	0.460
Non-Black	175	42.8	43	38.7	32	36.4	250	41.1	
Education									
Elementary	78	19.1	26	23.4	33	37.5	37	22.5	< 0.001
Secondary	202	49.4	69	62.2	48	54.5	319	52.5	
Higher education	129	31.5	16	14.4	7	8.0	152	25.0	
School delay									
No	267	65.3	76	68.5	57	64.8	400	65.8	0.802
Yes	142	34.7	35	31.5	31	35.2	208	34.2	
Work									
No	192	48.7	52	47.7	54	62.1	298	50.5	0.064
Yes	202	51.3	57	52.3	33	37.9	292	49.5	
Private health insurance									
No	283	69.2	89	80.2	69	79.3	441	72.7	0.023
Yes	126	30.8	22	19.8	18	20.7	166	27.3	
Access to information									
Knew about self-testing									
No	165	42.7	52	46.8	35	39.8	252	43.1	0.591
Yes	221	57.3	59	53.2	53	60.2	333	56.9	
Knew about PrEP									
No	100	24.6	16	14.4	33	37.5	149	24.6	< 0.001
Yes	307	75.4	95	85.6	55	62.5	457	75.4	
Risk perception									
Used condoms during first intercourse ^a^									
No	229	58.3	49	45.8	51	58.6	329	56.0	0.061
Yes	164	41.7	58	54.2	36	41.4	258	44.0	
Risk self-assessment ^a,b^									
1 to 6 (low)	320	82.3	77	72.0	78	90.7	475	81.6	< 0.001
≥ 7 (high)	69	17.7	30	28.0	8	9.3	107	18.4	
Access to prevention									
Used PEP or tested for HIV previously									
No	125	30.6	42	37.8	42	47.7	209	34.4	0.006
Yes	284	69.4	69	62.2	46	52.3	399	65.6	
Risk for HIV									
Casual partnership ^a^									
No	110	27.0	23	20.7	36	40.9	169	27.9	0.006
Yes	297	73.0	88	79.3	52	59.1	437	72.1	
Anal sex without a condom ^a^									
No	54	13.2	13	11.7	16	18.2	83	14	0.376
Yes	355	86.8	98	88.3	72	81.8	525	86	
History of STIs									
No	319	78.0	83	74.8	76	86.4	478	78.6	0.122
Yes	90	22.0	28	25.2	12	13.6	130	21.4	
Contexts of greater vulnerability to HIV									
Sexual work ^a,c^									
No	335	81.9	93	83.8	71	80.7	499	82	0.842
Yes	74	18.1	18	16.2	17	19.3	109	18	
Use of marijuana ^a,d^									
No	271	67.4	80	72.7	47	55.3	398	66.7	0.032
Yes	131	32.6	30	27.3	38	44.7	199	33.3	
Use of other drugs ^a,d^									
No	356	88.8	102	92.7	73	85.9	531	89.1	0.296
Yes	45	11.2	8	7.3	12	14.1	65	10.9	

CO: community organization; CTA: testing and counseling center; PEP: post-exposure prophylaxis; STI: sexually transmitted infection; PrEP: pre-exposure prophylaxis.

a Last six months.

b Question: How do you assess your risk of becoming infected with HIV, with 1 being the lowest risk and 10 being the highest?

c Refers to sex work or exchanging sex for money or gifts.

d Used during sex.

Adolescents from both COs were associated with living in regions with lower HDI. This association was stronger for the CO-outskirts: ORa = 0.82; 95%CI 0.78–0.86), whose average HDI was lower than the global average of the three organizations/services (0.721 x 0.775). In the CO-center, the association with lower HDI was marginal (ORa = 0.97; 95%CI 0.94–1.00) ( Tables 1 and 2 ).

**Table 2.** Indicators of access to PrEP in adolescent men who have sex with men, *travestis* , transgender women, and transfeminine people by institution. PrEP1519 Study.

**Characteristics**	**CTA-center**	**CO-center**	**CO-outskirts**	**Total**	**p**
Distance from home to institution (km)					
Mean (95%CI)	15.0 (12.8–17.2)	13.7 (12.2–15.3)	7.8 (6.1–9.5)	14.2	0.012
Median (IQ) ^a^	12.5 (6.3–19.8)	12.8 (8.1–18.1)	6.4 (2.1–10.0)	11.5 (5.7–18.5)	
Housing HDI ^b^					
Mean (95%CI)	0.787 (0.778–0.796)	0.770 (0.752–0.787)	0.721 (0.709–0.733)	0.775	< 0.001
Median (IQ) ^a^	0.777 (0.706–0.861)	0.765 (0.700–0.842)	0.723 (0.699–0.750)	0.765 (0.702–0.842)	
Demand creation - n (%)					
Community ^c^	217 (59.5)	84 (79.2)	36 (46.8)	337 (61.5)	< 0.001
Within the service ^d^	148 (40.5)	22 (20.8)	41 (53.2)	211 (38.5)	
Time to start PrEP ^e^ - n (%)					
Same day	174 (42.5)	26 (23.4)	44 (50.6)	244 (40.0)	< 0.001
1 to 7 days	68 (16.6)	35 (31.5)	20 (23.0)	123 (20.0)	
≥ 8 days	167 (40.8)	50 (45.0)	23 (26.4)	240 (40.0)	

HDI: Human Development Index; 95%CI: 95% confidence interval; CO: community organizations; CTA: testing and counseling center; PrEP: pre-exposure prophylaxis.

a Interquartile range.

b Human Development Index by Human Development Unit for residents of São Paulo and municipal HDI for residents of other municipalities.

c Social and virtual places.

d Spontaneous demand and demand referred to by other institutions.

e Time between first contact with demand creation and PrEP prescription.

The increased chance of initiating PrEP between 1 and 7 days after the first contact with a peer educator was also common to adolescents in both COs, being 2.91 times in the CO-outskirts (95%CI 1.22–6.92) and 1.91 times in the CO-center (95%CI 1.10–3.31). Furthermore, in the CO-outskirts, same-day PrEP initiation ( Table 2 ) occurred in a higher proportion (50.6%) than in CTA-center (42.5%) and CO-center (23.4%). This difference (data not shown) remained in an adjusted analysis comparing CO-outskirts and CO-center (p = 0.003) but was similar (p = 0.071) between CO-outskirts and CTA-center ( Table 3 ). This result may be related to demand creation strategies, which in CO-outskirts and CTA-center occurred predominantly within the institutions (53.2% and 40.5%, respectively), through RDS, in the case of CO-outskirts, and spontaneous demand, in the case of CTA-center. In CO-center, on the other hand, 79.2% of the first contacts were outreach.

A shorter distance from home was ( Table 3 ) associated with CO-outskirts (ORa = 0.82; 95%CI 0.78–0.86). In this institution ( Table 2 ), the average distance between the organization and the home was 7.8 km (95%CI 6.1–9.5), approximately half that of the central institutions (CTA-center = 15.0 km; 95%CI 12.8–17.2; CO-center = 13.7 km; 95%CI 12.2–15.3). Adolescents initiating PrEP in the CO-outskirts were also 3.06 times more likely (95%CI 1.63–5.75) to be aged 15–17 compared with CTA-center.

Adolescents who initiated PrEP in the CO-outskirts and CO-center also had a profile of HIV vulnerability specific to each organization ( Table 3 ). In the center organization, when compared with CTA, adolescents presented lower vulnerability to HIV, given the increased chance of having a greater perception of the risk of infection (condom in the first intercourse: ORa = 1.83; 95%CI 1.15–2.90; high self-assessed risk: ORa = 2.02; 95%CI 1.18–3.44) and greater prior knowledge about PrEP (ORa = 2.08; 95%CI 1.10–3.91). This increased chance of indicating a high-risk perception in the CO-center was also observed compared with adolescents from the CO-outskirts (data not shown, ORa = 6.59; 95%CI 2.30–18.89). In CO-outskirts, in contrast, there were threshold associations for adolescents who reported lower access to prevention (use of previous HIV testing and PEP: ORa = 1.89; 95%CI 0.99–3.60) and low self-assessment of high risk (ORa = 0.42; 95%CI 0.17–1.03).

**Table 3.** Adjusted odds ratio (ORa) and 95% confidence interval (95%CI) for PrEP start in the CO-center and CO-outskirts, compared with CTA-center. Adolescent men who have sex with men, *travestis* , transgender women, and transfeminine people. PrEP 1519 Study.

**Characteristics**	CO-center					CO-outskirts			
	ORa	95%CI		p		ORa	95%CI		p
		Min	Max				Min	Max	
Distance from home to institution (km)	0.98	0.95	1.01	0.229		0.82	0.78	0.86	< 0.001
Housing HDI ^a^	0.97	0.94	<1.00	0.049		0.82	0.78	0.86	< 0.001
Age group									
15 to 17 years	1.64	0.98	2.74	0.061		2.89	1.53	5.45	< 0.001
18 and 19 years	1					1			
Time to start PrEP ^b^									
Same day	0.47	0.27	0.81	0.007		1.9	0.95	3.82	0.071
1 to 7 days	1.91	1.10	3.31	0.021		2.91	1.22	6.91	0.016
≥ 8 days	1					1			
Used PEP or HIV test previously									
No	1.49	0.92	2.41	0.110		1.89	0.99	3.60	0.052
Yes	1					1			
Used condoms during first intercourse									
Yes	1.83	1.15	2.90	0.010		1.27	0.69	2.35	0.450
No	1					1			
Risk self-assessment ^c^									
≥ 7 (high)	2.02	1.18	3.44	0.010		0.42	0.17	1.03	0.059
1 to 6 (low)	1					1			
Knew about PrEP									
Yes	2.01	1.10	3.91	0.023		1.14	0.56	2.32	0.726
No	1					1			

CO: community organization; HDI: Human Development Index; CTA: testing and counseling center; PrEP: pre-exposure prophylaxis; PEP: post-exposure prophylaxis.

a Human Development Index by the Human Development Unit for residents of the city of São Paulo and a municipal HDI for residents of other municipalities.

b Time between first contact with demand creation and prescription of PrEP.

c Question: How do you assess your risk of becoming infected with HIV, with 1 being the lowest risk and 10 being the highest?

None of the characteristics related to sexual practices and being in contexts of greater vulnerability for prevention (prostitution and drug use) contributed to differentiating adolescents from COs and CTA-center.

While skin color/race was not associated with adolescents from COs when included in the model (data not shown), this variable altered the association with HDI, age, and risk self-assessment. The changes were more significant in the CO-center, losing the association with HDI (p = 0.071) and increasing the chance of starting PrEP between 15 and 17 years of age (p = 0.044). In the CO-outskirts, the association occurred with the lowest high-risk self-assessment (p = 0.049).

## DISCUSSION

We showed that the provision of PrEP in a civil society organization promoted better timely access to socially vulnerable adolescents. Particularities of the locations and organizations were essential in favor, in the outskirts, in a CO that worked with the general population, the inclusion of younger adolescents living in the community and with less access to prevention; and, in the center, in an LGBTQIAPN+ institution, more informed adolescents, with greater risk perception and coming from different parts of the city. The provision in a community context, however, did not favor the inclusion of a larger proportion of adolescents with sexual practices of greater risk for HIV.

Different ways of offering PrEP close to the community have shown positive effects for LGBTQIAPN+ people, such as those provided in pharmacies, by peers, or in primary care ^4^^,^^ = 5 *et al.* , 2018^^,^^23^ . We chose a model that valued an offer legitimized by organizations resulting from the actions of citizens, involving peers and prescription by nurses. This type of offer has been associated with a greater capacity to reach populations affected by HIV, including adolescents, increase prophylaxis initiation rates, and improve the PrEP continuum in different contexts ^8^^,^^10^^,^^23^ . These results are attributed in the literature to greater proximity to the values and needs of the population, convenience of access, and adoption of more specific protocols closer to comprehensive care, resulting in a greater capacity to deal with structural barriers, such as cisheteronormativity, stigma, violence and/or organizational and professional unpreparedness ^6^^,^^24^^,^^25^ . These factors have made it difficult, especially in more conventional health services, to create a more welcoming environment for prescribing PrEP, compromising, among other aspects, the disclosure of sexual orientation and the expression of the intention to use preventive methods ^6^^,^^10^ .

Furthermore, the PrEP protocol adopted in the present study focused the entire care process on nursing and peer educators, in contrast to the conventional service, in which prophylaxis prescription involved different stages and only healthcare professionals. This may have favored a more personalized and friendly offer. In this sense, studies have shown that nursing has a high acceptance of PrEP due to less judgment and greater proximity to individuals ^11^^-^^13^ . The same is highlighted for peer educators ^6^^,^^23^ , as they reduce embarrassment for young people, reinforce their protagonism, and facilitate the sharing of experiences about sexuality, prevention, and care.

A particular profile of access and characteristics of adolescents was also observed for each CO. These differences probably reflected the specificities of each institution’s location and type of activity. Including younger adolescents with less access to prevention in CO-outskirts may have been favored by the profile of the local population ^19^ and the type of activity of the CO aimed at the general population. This last aspect may have reduced the adolescents’ fear of suffering from the stigma associated with HIV and the LGBTQUIAPN+ community. In Vancouver and London ^6^^,^^25^ , for example, fear of stigma was one of the main reasons for MSM not to seek a service in the community or close to their area of residence. Similarly, the inclusion of adolescents more concerned about HIV in the CO-center may have been favored by the fact that it is an LGBTQUIAPN+ organization, which tends to bring together people who are more engaged and aware of the issue. In addition, the central region of the city is historically recognized as less hostile to expressions of non-hegemonic sexualities and genders ^18^ , serving as a hub for those seeking environments more inclusive of LGBTQUIAPN+ culture.

These results reinforce that services with different characteristics can strengthen universalization and equity in PrEP ^4^^,^^5^ , especially in regions with lower HDI ^1^^,^^26^ .

The absence of differences in the profile of vulnerability to HIV between the institutions analyzed contrasts with other studies that identified a higher frequency of risk in community strategies ^10^^,^^27^ . While there are differences between the services, it is possible that the outreach strategies for creating demand ^20^ , developed in the three institutions, contributed to making the clientele profile more homogeneous. Another aspect to consider is that the sample size may have made identifying some differences difficult. Finally, it is worth highlighting that the adolescents included in the study had a slightly higher risk prevalence than the average PrEP users in Brazilian public network services ^28^ .

No differences were observed for the inclusion of aTTrans either, although the proportion of this population was approximately 40% higher in the CO-outskirts than in other services. We attribute this higher number to intensifying strategies aimed at CO-outskirts, such as discussion groups and gender affirmative actions. However, the barriers to access for aTTrans are multiple and interconnected, ranging from fear of HIV testing to structural aspects ^29^ , limiting the scope of actions that partially address the problem.

In the same vein, we highlight race/ethnicity, which, although not associated with adolescents in the COs, influenced the association with other critical access-related issues, such as HDI, risk perception, and age at which PrEP was initiated. This reinforces the need to integrate the diversity of race/ethnicity that exists in Brazil into preventive strategies (e.g., including Black people in health teams and prioritizing this community in interventions), responding to structural racism.

The results of this study should be interpreted considering their limitations. Uncontrolled contextual factors, such as safety, transportation, or violence related to gender and/or sexual orientation, can directly influence access and define the characteristics of an institution’s clientele. It should be noted, however, that the results were observed from COs with substantially different features and that key activities to promote access were equally developed in the community and in conventional services, such as creating demand, training professionals, and financial support for transportation. Additionally, we included variables in the analysis that reflect the context more comprehensively, such as HDI and distance between home and service.

In conclusion, by approaching the territory and civil society organizations, community provision can be strategic for prevention policies, especially when incorporating new technologies. This can favor the expansion of equitable access to PrEP for adolescents who are at the beginning of their sexual trajectory and learning about self-care.
