# Supplementary material for: Prep provision in community organizations: a comparative study with conventional services
Source: Rev Saude Publica. 2024 Oct 11;58(Supl 1):9s. doi: 10.11606/s1518-8787.2024058005914 (PMC11573378; doi:10.11606/s1518-8787.2024058005914)
Supplement: Translation

# Translation: Oferta de prep em organizações comunitárias: estudo comparativo com serviços convencionais

**Authors:** Alexandre Grangeiro, Paula Andrea Massa, Maria Mercedes Escuder, Eliana Miura Zucchi, Eliane Aparecida Sala, Eduardo Araujo de Oliveira, Raphaela Fini, Inês Dourado, Laio Magno, Beo Oliveira Leite, Katia Bruxvoort, Sarah MacCarthy, Marcia Thereza Couto, Maria Fernanda Tourinho Peres

**Affiliations:** I Universidade de São Paulo. Faculdade de Medicina. São Paulo, SP, Brasil Universidade de São Paulo Faculdade de Medicina SP São Paulo Brasil; II Secretaria de Estado da Saúde de São Paulo. Instituto de Saúde. São Paulo, SP, Brasil Secretaria de Estado da Saúde de São Paulo Instituto de Saúde SP São Paulo Brasil; III Universidade Católica de Santos. Programa de Pós-Graduação em Saúde Coletiva. Santos, SP, Brasil Universidade Católica de Santos Programa de Pós-Graduação em Saúde Coletiva SP Santos Brasil; IV Universidade Federal da Bahia. Instituto de Saúde Coletiva. Salvador, BA, Brasil Universidade Federal da Bahia Instituto de Saúde Coletiva BA Salvador Brasil; V Universidade do Estado da Bahia. Departamento de Ciências da Vida. Salvador, BA, Brasil Universidade do Estado da Bahia Departamento de Ciências da Vida BA Salvador Brasil; VI University of Alabama at Birmingham. Birmingham, AL, United States of America University of Alabama at Birmingham AL Birmingham United States of America

**Keywords:** HIV, Profilaxia Pré-Exposição, Organização Comunitária, Adolescente, Estudo de Intervenção

## Abstract

**OBJETIVO:**

Avaliar se adolescentes de minorias sexuais que iniciaram a profilaxia pré-exposição sexual (PrEP) em organizações comunitárias (OC) apresentam maior vulnerabilidade social e ao HIV em comparação com adolescentes em PrEP de um serviço de saúde convencional. Além disso, avaliar se esses adolescentes tiveram um acesso mais oportuno à profilaxia.

**MÉTODOS:**

Estudo demonstrativo da efetividade de PrEP, realizado na cidade de São Paulo, em duas OC, localizadas no centro (OC-centro) e na periferia (OC-periferia), e em um serviço convencional de testagem para o HIV (CTA-centro). Foram elegíveis para PrEP, entre 2020 e 2022, adolescentes homens cisgêneros que fazem sexo com homens (aHSH), travestis, mulheres transexuais e pessoas transfemininas (aTTrans), de 15 a 19 anos, HIV-negativos e com práticas de maior risco para o HIV. Indicadores de acesso oportuno e de vulnerabilidades dos adolescentes iniciando PrEP nas OC foram analisados, tendo por referência o CTA-centro e empregando regressão logística multinomial.

**RESULTADOS:**

608 adolescentes iniciaram PrEP nas OC e CTA-Centro. Adolescentes das OC estiveram associados a um menor tempo de início de PrEP (1–7 dias; OC-periferia: ORa = 2,91; IC95% 1,22–6,92; OC-centro: ORa = 1,91; IC95% 1,10–3,31); e a um menor IDH de moradia (OC-centro: ORa = 0,97; IC95% 0,94–1,00; OC-periferia: ORa = 0,82; IC95% 0,78–0,86). Na OC-periferia houve aumento na chance de os adolescentes serem mais jovens (ORa = 3,06; IC95% 1,63–5,75) e morarem mais próximos ao serviço (ORa = 0,82; IC95% 0,78–0,86, média 7,8 km). Enquanto adolescentes da OC-centro estiveram associados ao maior conhecimento prévio de PrEP (ORa = 2,01; IC95% 1,10–3,91) e a alta percepção de risco (ORa = 2,02; IC95% 1,18–3,44). Não estiveram associadas aos adolescentes das OC as práticas sexuais de maior risco e as situações de vulnerabilidade ao HIV.

**CONCLUSÕES:**

A oferta de PrEP nas OC facilitou o acesso de adolescentes vulnerabilizados e pode contribuir para reduzir inequidades.

## INTRODUÇÃO

A ampliação do uso da profilaxia pré-exposição sexual (PrEP) para pessoas em maior vulnerabilidade social e econômica ao HIV é um desafio ao cumprimento das metas de eliminação da epidemia de HIV até 2030 ^1^ . Isso tem levado a diversas iniciativas para simplificar a oferta da profilaxia, como a prescrição por diferentes profissionais, a oferta fora dos serviços de saúde e o uso de tecnologias da comunicação ^2^^-^^4^ . Apesar disso, a maior parte das ofertas de PrEP permanece centrada em médicos e em serviços especializados, tanto em países de alta renda, como de baixa e média renda ^3^^,^^4^ . Persistem, também, importantes barreiras de acesso aos serviços ^5^^,^^6^ , como o estigma, o receio de revelação da orientação sexual e identidade de gênero em serviços não amigáveis, a inadequação entre a oferta de PrEP e o cotidiano das pessoas. Além disso, é reduzido o acesso de adolescentes à PrEP, levando a uma presença restrita dessa população nos serviços e a estudos de eficácia e efetividade ^7^ . Por isso, é necessário produzir conhecimentos mais apurados e adequados às necessidades dessa população.

Muitos estudos avaliaram alternativas para ofertar PrEP, com o reconhecimento que inexiste uma única forma para abranger a diversidade de contextos e populações prioritárias 4 ^,^^5^ . Nessa direção, a oferta de PrEP na comunidade tem se mostrado efetiva, com experiências que exploram diferentes tipos de serviços e profissionais responsáveis pela prescrição. Na Tailândia e no Vietnã ^8^^,^^9^ , a prescrição comunitária para homens cisgênero que fazem sexo com homens (HSH) e mulheres transgênero foi realizada por pares treinados e identificados nas redes sexuais, melhorando o grau de identificação de pessoas em risco e a proporção de início da profilaxia. Na Namíbia ^10^ , a prescrição para mulheres adolescentes e jovens foi realizada na comunidade, por profissionais da medicina e enfermagem, com as usuárias escolhendo o local e hora para receber a prescrição. Isto se refletiu numa melhora da continuidade do uso, quando comparada a serviços de saúde convencionais.

A prescrição por diferentes profissionais também tem se associado à ampliação do acesso e a melhorias na cascata de PrEP. A enfermagem ganha destaque devido à aceitação dos usuários ^11^ , motivação para a prescrição ^12^^,^^13^ e inserção em atividades que favorecem a triagem e o seguimento da profilaxia ^4^ . A maior atuação da enfermagem vem acompanhada da reformulação de protocolos de PrEP para atuação da categoria ^3^^,^^14^ e de questionamentos sobre a permanência dos resultados positivos da atuação da enfermagem em serviços não especializados ^4^ .

No Brasil, a PrEP pode ser prescrita por diferentes profissionais, em serviços especializados, de atenção primária e consultórios privados, utilizando a diretriz nacional ^15^ e os medicamentos fornecidos pelo Sistema Único de Saúde (SUS). Apesar dessa amplitude, a cobertura da profilaxia permanece baixa e concentrada em adultos com maior status socioeconômico ^16^ . Por isso, em 2020, no âmbito do estudo PrEP1519, foi implantado, em São Paulo, um estudo demonstrativo para avaliar, em adolescentes, o acesso e a efetividade de ofertar a PrEP em duas organizações comunitárias (OC). No presente artigo, avaliamos se adolescentes homens cisgêneros que fazem sexo com homens (aHSH), travestis, mulheres transexuais e pessoas transfemininas (aTTrans), que utilizavam PrEP nessas duas OC, tinham maior vulnerabilidade social e ao HIV; e se tiveram um acesso mais oportuno à profilaxia, quando comparados aos usuários adolescentes de um serviço de saúde convencional.

## MÉTODOS

### O Estudo PrEP1519

Trata-se de um estudo demonstrativo de PrEP oral diária ^17^ , desenvolvido em Belo Horizonte, Salvador e São Paulo, com adolescentes de 15 a 19 anos, que se declaravam aHSH e aTTrans. A PrEP foi ofertada seguindo o protocolo brasileiro ^15^ , que define como critérios principais de prescrição ter o teste anti-HIV negativo e ter um maior risco ou vulnerabilidade ao HIV; e, como critério de exclusão, o comprometimento da função renal ou não ter suficiente compreensão para a adesão e permanência em PrEP. Em São Paulo, o estudo foi iniciado em fevereiro de 2019, em um dos centros de testagem e aconselhamento mais tradicionais da cidade, localizado na região central (CTA-centro), bairro da Sé, com prescrição por equipe médica e suporte de aconselhadores. O CTA-centro, no presente estudo, foi definido como “serviço de saúde convencional”. Em 2020, o estudo foi ampliado, exclusivamente na cidade de São Paulo, para ofertar PrEP em duas OC, com equipes extramuros. O objetivo foi comparar essa oferta na comunidade com o serviço convencional de saúde, assumindo o pressuposto de que ofertar a profilaxia em um contexto mais amigável e próximo da comunidade propiciaria a redução das barreiras de acesso para adolescentes não incluídos, regularmente, nos serviços de saúde.

### Organizações Comunitárias

Definimos como organizações comunitárias aquelas que resultam da ação coletiva de cidadãos em prol de interesses e melhorias para uma população. Assim, para permitir uma melhor comparação, selecionamos uma OC que estava inserida numa região similar ao do CTA-centro; e outra numa área substancialmente oposta em relação à concentração de riquezas, características populacionais e condições de acesso à saúde. Para essa escolha foram realizadas pesquisas formativas ^18^ nas regiões leste e central de São Paulo. No centro, bairro da Bela Vista, área contígua ao do CTA-centro, foi identificada uma OC (OC-centro) que atua no suporte social e defesa de direitos LGBTQIAPN+; e, no extremo leste, bairro de Cidade Tiradentes, uma organização (OC-periferia) que desenvolve ações para a população geral, visando responder aos desafios que afetam a periferia.

Os três bairros que abrigam as organizações/serviço do estudo se diferem ^19^ , entre outros aspectos, pelas proporções de pessoas negras (21,6% - Bela Vista; 38,3% - Sé; 56,1% - Cidade Tiradentes); com idade entre 0 e 29 anos (27,2% - Bela Vista; 39,2% - Sé; 48,5% - Cidade Tiradentes); que residem num raio de 1 km das estações de trem/metro (86,4% - Sé; 54,7% - Bela Vista; 0,0% - Cidade Tiradentes); e na renda média mensal do trabalho (R$4,403,34 - Bela Vista; R$4.197,02 – Sé; R$2.654,42 - Cidade Tiradentes).

### Protocolo para Ofertar PrEP nas Organizações Comunitárias

O protocolo está detalhado no Quadro 1 , assim como as diferenças em relação ao serviço convencional. Em síntese, cada OC recebeu uma equipe extramuro, composta por enfermeiros e educadores-pares selecionados na comunidade. Essa equipe era responsável por todas as atividades de criação de demanda, cuidado clínico, vinculação, adesão e retenção. Um grupo de profissionais de medicina, psicologia e serviço social foi constituído como uma equipe de apoio, para dar suporte na discussão de caso e no acompanhamento de adolescentes que exigiam abordagens mais complexas ou interdisciplinares.

Estratégias de criação de demanda e vinculação foram desenvolvidas para identificar adolescentes com maior risco para o HIV em ambientes de sociabilidade, sendo elas: “ *na comunidade* ”, envolvendo locais de encontro, redes sociais e aplicativos para busca de parceiros; e “ *dentro das instituições* ”, com a identificação de adolescentes na clientela e/ou referido por outras instituições. Essas estratégias, desenvolvidas principalmente por educadores-pares, abrangiam, igualmente, as OC e CTA-centro ^20^ . Na OC-periferia, adicionalmente, foi utilizada uma estratégia empregada em pesquisas com populações de difícil acesso, denominada amostragem dirigida pelo participante ^21^ ou, em inglês, *respondent driven sampling* (RDS). Nela, um participante é incentivado a convidar outros três potenciais participantes, desencadeando sucessivas “ondas”.

Adolescentes identificados na criação de demanda e que manifestavam interesse em PrEP eram, então, envolvidos nas estratégias de vinculação. Nelas, o adolescente era acompanhado por um educador-par, por meio virtual ou presencial, até o momento de chegada à organização.

Os adolescentes que chegavam às organizações eram triados, iniciavam PrEP e eram seguidos clinicamente de acordo com o protocolo do Ministério da Saúde, simplificado e detalhado para aplicação num contexto comunitário e prescrição por enfermagem ( Quadro 1 ). Para as infecções sexualmente transmissíveis (IST), foi utilizada a abordagem sindrômica e etiológica, com diagnóstico e prescrição do tratamento realizada na instituição e retirada/aplicação de medicamentos em um serviço da rede pública.

**Quadro 1.** Protocolo de atendimento para oferta de PrEP em organizações comunitárias. Estudo PrEP 1519.

**Processo**	**Oferta comunitária**	**Diferença em relação ao serviço convencional** ^a^
**Equipe**	Equipe extramuro atuando em cada organização comunitária: um profissional de enfermagem, responsável pela prescrição, manejo de IST, eventos adversos e aconselhamento; e dois educadores de par, responsáveis pela criação de demanda, acolhimento, vinculação, retenção e grupos de discussão. Equipe de apoio para as OC: profissional de medicina, assistência social e psicologia, responsáveis pela discussão de caso e seguimento de casos com maior complexidade.	Profissionais de recepção, enfermagem, técnico de enfermagem, aconselhadores e medicina
**Treinamento**	Formação continuada, com reuniões mensais, com temas relacionados à sexualidade, LGBTQUIAPN+ e prevenção. Dinâmica: discussão com convidados externos e discussão de casos.	Não realizado
**Espaço físico**	Compartilhado, composto por: consultório clínico, com área de coleta e sala de aconselhamento	Espaço de uso exclusivo
**Horário de funcionamento**	Dias úteis: tarde e noite; sábados: tarde	Dias úteis: manhã e tarde
**Criação de demanda**	Ações realizadas fundamentalmente por educador par, que busca identificar pessoas que possam se beneficiar de PrEP, esclarecer os benefícios e acompanhá-los até o serviço, quando necessário. Essas ações foram realizadas em: locais presenciais de sociabilidade e festas; espaços virtuais em aplicativos de encontro de parceiros e outras redes sociais, como Instagram. Foi denominado como demanda espontânea quando um participante indicou o serviço a outros ou quando eles foram indicados por um serviço, instituição ou profissional de saúde. Na OC-periferia utilizamos, adicionalmente, a estratégia *respondent driven sampling* (RDS). Nela, adolescentes homens que fazem sexo com homens foram incentivados a convidarem outros três adolescentes para responder a uma pesquisa de comportamento e assim sucessivamente, formando uma “espécie de ondas”. O incentivo para o convite foi realizado por meio de uma ajuda de custo e kit de prevenção. Adolescentes elegíveis para PrEP, após responder a pesquisa de comportamento, eram convidados para iniciar PrEP, após aconselhamento.	Similiar, excetuando RDS
**Vinculação**	Acesso/primeira consulta: acompanhamento por meio virtual e presencial no deslocamento (navegação), se necessário. Consultas de seguimento: mínimo de 2 contatos virtuais nos primeiros 30 dias e 1 contato entre consultas. Todas as consultas: disponibilidade de apoio financeiro para transporte e alimentação. Profissional responsável: educador par.	Similar, realizado por profissional de saúde
**Teste rápido**	Trimestral: HIV e Sífilis; semestral hepatite C e B (sem imunização)	Similar
**Clamídia e gonorreia**	Semestral: anal, uretral, oral (material obtido por autocoleta)	Não disponível na rede pública
**Coleta de exames de segurança**	Função renal e hepática anual	Similar
**Manejo de eventos adversos**	Eventos graus 1 e 2: atendimento feito por enfermagem; evento grau 2: sem remissão de sintomas avaliado pelo profissional médico; e graus 3 e 4: encaminhamento a serviço de emergência.	Evento com grau 1 atendido por enfermagem/médico e o restante pelos profissionais médicos
**Alterações em exames laboratoriais**	Manejo definido segundo critérios DAIDS, com atendimento inicial por enfermagem. Referido para abordagem médica: eventos grau 2 com ausência de melhora; uso de medicações para doenças crônicas e/ou comorbidades; e evento graus 3/4 assintomático.	Evento com grau 1 atendido por enfermagem/médico e o restante pelos profissionais médicos
**Manejo de IST**	Diagnóstico sindrômico e etiológico e prescrição do tratamento, como retirada do medicamento na rede pública de saúde.	Diagnóstico sindrômico e etiológico e tratamento
**Adesão e retenção**	Navegação do cuidado com oferta de: teleatendimento, rodas de conversa, apoio virtual ou presencial para o acesso ao serviço e a outros serviços, acolhimento psicossocial feito por educador par e equipe de apoio para casos com maior complexidade (assistente social, psicólogo), Encaminhamento para a rede de atenção psicossocial, se necessário.	Acolhimento psicossocial, encaminhamento para rede de atenção à saúde
**Grupos e rodas de conversa**	Grupo para afirmação de gênero e inserção social de travestis e mulheres transexuais; Grupo para adolescentes homens que fazem sexo com homens e Grupos para adesão e vinculação. Características: abertos para adolescentes em PrEP e da comunidade, com periodicidade quinzenal, mediado por educador-par e profissional equipe de apoio.	Não realizado

OC: organização comunitária; IST: infecção sexualmente transmissível; PrEP: profilaxia pré-exposição sexual; DAIDS: Classificação de Eventos Adversos da Divisão de Aids dos Estados Unidos da América; PCDT: Protocolo Clínico e Diretrizes Terapêuticas; CTA: centro de testagem e aconselhamento.

a Baseado no protocolo adotado no CTA - centro, da Secretaria Municipal de Saúde e no Protocolo Clínico e Diretrizes Terapêuticas do Ministério da Saúde.

Após a prescrição inicial de PrEP, o adolescente era contatado pelos educadores pares e equipe de apoio, por meio virtual, como suporte à adesão, à retenção ou a outras demandas psicossociais. O suporte em PrEP envolvia, adicionalmente, acolhimento, grupos de discussão e encaminhamento para serviços. Para as aTTrans, foi criado um grupo específico para apoiar o processo de cuidado, abordando temas como transfobia, afirmação de gênero e suporte social. O fluxo de atendimento pode ser observado na Figura 1.

**Figura 1.** Componentes e fluxo de atendimento na oferta de PrEP em organizações comunitárias. Estudo PrEP 1519.

### População e Período de Estudo

Foram incluídos, no presente estudo, os adolescentes que receberam pelo menos uma prescrição de PrEP em algum dos três serviços do estudo, entre fevereiro de 2020 e fevereiro de 2022.

### Pressupostos e Variáveis

Para a seleção de variáveis, adotamos o pressuposto de que a oferta num ambiente comunitário, comparada ao serviço convencional (CTA-centro), aumentaria a chance do início de PrEP e dos adolescentes terem as seguintes características:

1. Acesso oportuno: residirem mais próximos da organização e terem a prescrição inicial de PrEP em até sete dias após o primeiro contato com os educadores-pares. A distância do local de moradia foi considerada com indicador de acesso oportuno pelo pressuposto de que a oferta na comunidade reduziria o estigma e, assim, propiciaria o acesso prioritário de pessoas residindo localmente e no menor tempo possível.
2. Maior vulnerabilidade social: inclusão de adolescentes provenientes de regiões com menor Índice de Desenvolvimento Humano (IDH). Adotamos o IDH por considerar que ele discrimina melhor a vulnerabilidade social do que as características individuais. No caso de adolescentes, que compreendem uma faixa etária estreita, a escolaridade normalmente apresenta pequena variação, enquanto informações sobre renda familiar e/ou situação habitacional podem ser pouco conhecidas pelo adolescente.
3. Maior vulnerabilidade ao HIV: menor grau de informação (não conhecer previamente PrEP e autoteste); baixa autopercepção do risco (autoavaliação do risco ≤ 6, numa escada de 10, e sem uso do preservativo na primeira relação sexual); menor acesso à prevenção (nunca ter realizado a profilaxia pós-exposição sexual [PEP] ou teste HIV); adoção de práticas de maior risco (ter maior número de parceria casual, sexo anal sem camisinha e histórico de IST); e estar em contextos de vulnerabilidade para a prevenção (trabalho sexual, uso de maconha e de outras drogas no momento do sexo).

A distância moradia-instituição foi calculada no Open Source Geographic Information System, versão 3,32. O tempo de início de PrEP foi determinado pelas datas do primeiro contato com uma estratégia de criação de demanda e a prescrição inicial de PrEP, categorizada em: “mesmo dia”, “1 a 7 dias” e “≥ 8 dias”. Para a categorização do IDH, utilizamos, no município de São Paulo, o local de residência por Unidades de Desenvolvimento Humano (UDH), que agregam áreas homogêneas e contíguas, definidas com dados dos setores censitários ^22^ . Para outros municípios, a referência foi o IDH municipal.

As informações foram obtidas dos registros eletrônicos de atendimento em sistema online do estudo, implantado nas três instituições. Práticas, percepção e contexto de vulnerabilidade foram referentes aos três meses antecedentes à inclusão e, as demais, para toda a vida.

### Análise Estatística

Foi utilizada regressão logística multinomial, com intervalos de confiança de 95% (IC95%), para analisar a associação das variáveis de início oportuno, vulnerabilidade social e vulnerabilidade ao HIV com as OC, tendo como referência o CTA-centro. Todas as variáveis ( Tabelas 1 e 2 ) foram incluídas na análise, com exceção da escolaridade, por ser parâmetro incluído no IDH, e as estratégias de criação de demanda, por serem intrínsecos ao processo de trabalho das organizações/serviço. Variáveis com nível de significância de ≤ 0,1 na análise bivariada foram incluídas no modelo inicial multivariado, permanecendo, ao final, aquelas com nível de significância ≤ 0,05%. Dados faltantes foram excluídos. Análises foram realizadas no SPSS, versão 23.

Os participantes assinaram o Termo de Consentimento Livre e Esclarecido. Adolescentes entre 15 e 17 anos tiveram dispensa do consentimento parental, por sentença-alvará judicial (SP:1128747-15.2018.8.26.0100). O protocolo foi aprovado pela Comissão de Ética do Hospital das Clínicas da Faculdade de Medicina da Universidade de São Paulo, com parecer número 4.488.944.

## RESULTADOS

Iniciaram a PrEP 608 adolescentes, majoritariamente no CTA-centro (n = 409, 67,3%). O perfil sociodemográfico deles se diferenciou (p < 0,05), exclusivamente, pela predominância de pessoas mais jovens (15–17 anos, 42%) e com menor escolaridade (fundamental, 37,5%) na OC-periferia, assim como por uma maior proporção (30,8%) de adolescentes com plano privado de saúde no CTA-centro ( Tabela 1 ).

**Tabela 1.** Características de adolescentes homens que fazem sexo com homens, travestis, mulheres transexuais e pessoas transfemininas que iniciaram PrEP, em São Paulo, de fevereiro de 2019 a fevereiro de 2022. Estudo PrEP 1519.

**Características**	**CTA-centro**		**OC-centro**		**OC-periferia**		**Total**		
	n	%	n	**%**	n	%	n	%	p
Total	409	67,3	111	18,3	88	14,5	608	100	
Demográficas									
Gênero									
Homem cisgênero	378	92,4	104	93,7	77	87,5	559	91,9	0,231
Mulher transexual e travesti	31	7,6	7	6,3	11	12,5	49	8,1	
Faixa etária									
15 a 17	92	22,5	33	29,7	37	42,0	162	26,6	< 0,001
18 a 19	317	77,5	78	70,3	51	58,0	446	73,4	
Cor de pele									
Negra	234	57,2	68	61,3	56	63,6	358	58,9	0,460
Não negra	175	42,8	43	38,7	32	36,4	250	41,1	
Escolaridade									
Fundamental	78	19,1	26	23,4	33	37,5	37	22,5	< 0,001
Médio	202	49,4	69	62,2	48	54,5	319	52,5	
Superior	129	31,5	16	14,4	7	8,0	152	25,0	
Atraso escolar									
Não	267	65,3	76	68,5	57	64,8	400	65,8	0,802
Sim	142	34,7	35	31,5	31	35,2	208	34,2	
Trabalho									
Não	192	48,7	52	47,7	54	62,1	298	50,5	0,064
Sim	202	51,3	57	52,3	33	37,9	292	49,5	
Plano privado de saúde									
Não	283	69,2	89	80,2	69	79,3	441	72,7	0,023
Sim	126	30,8	22	19,8	18	20,7	166	27,3	
Acesso à informação									
Conhecia autoteste									
Não	165	42,7	52	46,8	35	39,8	252	43,1	0,591
Sim	221	57,3	59	53,2	53	60,2	333	56,9	
Conhecia PrEP									
Não	100	24,6	16	14,4	33	37,5	149	24,6	< 0,001
Sim	307	75,4	95	85,6	55	62,5	457	75,4	
Percepção do risco									
Uso preservativo na primeira relação ^a^									
Não	229	58,3	49	45,8	51	58,6	329	56,0	0,061
Sim	164	41,7	58	54,2	36	41,4	258	44,0	
Autoavaliação do risco ^a,b^									
1 a 6 (baixo)	320	82,3	77	72,0	78	90,7	475	81,6	< 0,001
≥ 7 (alto)	69	17,7	30	28,0	8	9,3	107	18,4	
Acesso à prevenção									
Usou PEP ou teste anti-HIV previamente									
Não	125	30,6	42	37,8	42	47,7	209	34,4	0,006
Sim	284	69,4	69	62,2	46	52,3	399	65,6	
Risco para o HIV									
Parceria casual ^a^									
Não	110	27,0	23	20,7	36	40,9	169	27,9	0,006
Sim	297	73,0	88	79,3	52	59,1	437	72,1	
Sexo anal sem preservativo ^a^									
Não	54	13,2	13	11,7	16	18,2	83	14	0,376
Sim	355	86,8	98	88,3	72	81,8	525	86	
História de IST									
Não	319	78,0	83	74,8	76	86,4	478	78,6	0,122
Sim	90	22,0	28	25,2	12	13,6	130	21,4	
Contextos de maior vulnerabilidade ao HIV									
Trabalho sexual ^a,c^									
Não	335	81,9	93	83,8	71	80,7	499	82	0,842
Sim	74	18,1	18	16,2	17	19,3	109	18	
Uso de maconha ^a,d^									
Não	271	67,4	80	72,7	47	55,3	398	66,7	0,032
Sim	131	32,6	30	27,3	38	44,7	199	33,3	
Uso de outras drogas ^a,d^									
Não	356	88,8	102	92,7	73	85,9	531	89,1	0,296
Sim	45	11,2	8	7,3	12	14,1	65	10,9	

OC: organização comunitária; CTA: centro de testagem e aconselhamento; PEP: profilaxia pós-exposição sexual; IST: infecção sexualmente transmissível; PrEP: profilaxia pré-exposição sexual.

a Últimos seis meses.

b Pergunta: Como você avalia seu risco de se infectar com o HIV, sendo 1 o risco mais baixo e 10 o mais alto?

c Refere ao trabalho sexual ou troca de sexo por dinheiro ou presentes.

d Uso durante a cena de sexo.

Adolescentes das duas OC estiveram associados à moradia em regiões com menor IDH. Essa associação foi mais intensa para a OC-periferia: ORa = 0,82; IC95% 0,78–0,86), cujo IDH médio foi inferior à média global das três organizações/serviço (0,721 x 0,775). Na OC-centro, a associação com menor IDH foi marginal (ORa = 0,97; IC95% 0,94–1,00) (Tabelas 2 e 3).

**Tabela 2.** Indicadores de acesso à PrEP em adolescentes homens que fazem sexo com homens, travestis, mulheres transexuais e pessoas transfemininas por instituição. Estudo PrEP1519.

**Características**	**CTA-centro**	**OC-centro**	**OC-periferia**	**Total**	**p**
Distância moradia-instituição (km)					
Média (IC95%)	15,0 (12,8–17,2)	13,7 (12,2–15,3)	7,8 (6,1–9,5)	14,2	0,012
Mediana (IQ) ^a^	12,5 (6,3–19,8)	12,8 (8,1–18,1)	6,4 (2,1–10,0)	11,5 (5,7–18,5)	
IDH de moradia ^b^					
Média (IC95%)	0,787 (0,778–0,796)	0,770 (0,752–0,787)	0,721 (0,709–0,733)	0,775	< 0,001
Mediana (IQ) ^a^	0,777 (0,706–0,861)	0,765 (0,700–0,842)	0,723 (0,699–0,750)	0,765 (0,702–0,842)	
Criação de demanda - n (%)					
Comunidade ^c^	217 (59,5)	84 (79,2)	36 (46,8)	337 (61,5)	< 0,001
Dentro do serviço ^d^	148 (40,5)	22 (20,8)	41 (53,2)	211 (38,5)	
Tempo para iniciar PrEP ^e^ - n (%)					
Mesmo dia	174 (42,5)	26 (23,4)	44 (50,6)	244 (40,0)	< 0,001
1 a 7 dias	68 (16,6)	35 (31,5)	20 (23,0)	123 (20,0)	
≥ 8 dias	167 (40,8)	50 (45,0)	23 (26,4)	240 (40,0)	

IDH: Índice de Desenvolvimento Humano; IC95%: intervalo de confiança de 95%; OC: organizações comunitárias; CTA: centro de testagem e aconselhamento; PrEP: profilaxia pré-exposição sexual.

a Intervalo interquartil.

b Índice de Desenvolvimento Humanos por Unidade de Desenvolvimento Humano, para residentes na cidade de São Paulo, e IDH municipal para residentes em outros municípios.

c Locais de sociabilidade e virtual.

d Demanda espontânea e referida por outras instituições.

e Tempo ente primeiro contato com criação de demanda e prescrição de PrEP.

Também foi comum aos adolescentes das duas OC o aumento na chance de iniciar PrEP entre 1 e 7 dias após o primeiro contato com educador par, na ordem de 2,91 vezes na OC-periferia (IC95% 1,22–6,92) e de 1,91 vezes na OC-centro (IC95% 1,10–3,31). Além disso, na OC-periferia, o início de PrEP no mesmo dia (Tabela 2) ocorreu numa maior proporção (50,6%) do que no CTA-centro (42,5%) e OC-centro (23,4%). Essa diferença (dados não mostrados) se manteve numa análise ajustada, comparando OC-periferia e OC-centro (p *=* 0,003), porém, se mostrou similar (p *=* 0,071) entre OC-periferia e CTA-centro (Tabela 3). É possível que esse resultado esteja relacionado às estratégias de criação de demanda, que na OC-periferia e CTA-centro ocorreram predominantemente dentro das instituições (53,2% e 40,5%, respectivamente), por meio da RDS, no caso da OC-periferia, e da demanda espontânea, no CTA-centro. Na OC-centro, opostamente, 79,2% dos primeiros contatos foram extramuros.

Uma menor distância da moradia esteve ( Tabela 3 ) associada à OC-periferia (ORa = 0,82; IC95% 0,78–0,86). Nessa instituição ( Tabela 3 ), a distância média organização-moradia foi de 7,8 km (IC95% 6,1–9,5), cerca da metade das instituições centrais (CTA-centro = 15,0 km; IC95% 12,8–17,2; OC-centro = 13,7 km; IC95% 12,2–15,3). Adolescentes iniciando PrEP na OC-periferia tiveram, também, uma chance 3,06 vezes maior (IC95% 1,63–5,75) de terem idade entre 15 e 17 anos, em comparação ao CTA-centro.

Os adolescentes que iniciaram PrEP na OC-periferia e na OC-centro apresentaram, adicionalmente, um perfil de vulnerabilidade ao HIV particular a cada organização ( Tabela 3 ). Na organização do centro, quando comparado ao CTA, os adolescentes apresentaram uma menor vulnerabilidade ao HIV, dado o aumento na chance de terem maior percepção do risco de infecção (preservativo na primeira relação: ORa = 1,83; IC95% 1,15–2,90; alta autoavaliação de risco: ORa = 2,02; IC95% 1,18–3,44) e maior conhecimento prévio sobre PrEP (ORa = 2,08; IC95% 1,10–3,91). Esse aumento da chance de indicar uma percepção de alto risco na OC-centro também foi observado na comparação com adolescentes da OC-periferia (dados não mostrados, ORa = 6,59; IC95% 2,30–18,89). Na OC-periferia, em contraste, houve associações limiares para adolescentes que relataram menor acesso à prevenção (uso de teste HIV e PEP prévios: ORa = 1,89; IC95% 0,99–3,60) e baixa autoavaliação de alto risco (ORa = 0,42; IC95% 0,17–1,03).

**Tabela 3.** Odds ratio ajustado (ORa) e intervalo de confiança de 95% (IC95%) para início de PrEP na OC-centro e OC-periferia, comparado ao CTA-centro. Adolescentes homens que fazem sexo com homens, travestis, mulheres transexuais e pessoas transfemininas. Estudo PrEP 1519.

**Características**	OC-centro					OC-periferia			
	ORa	IC95%		p		ORa	IC95%		p
		Min	Max				Min	Max	
Distância moradia-instituição (km)	0,98	0,95	1,01	0,229		0,82	0,78	0,86	< 0,001
IDH de moradia ^[Table-fn tfn08]a^	0,97	0,94	<1,00	0,049		0,82	0,78	0,86	< 0,001
Faixa etária									
15 a 17 anos	1,64	0,98	2,74	0,061		2,89	1,53	5,45	< 0,001
18 e 19 anos	1					1			
Tempo para iniciar PrEP ^b^									
Mesmo dia	0,47	0,27	0,81	0,007		1,9	0,95	3,82	0,071
1 a 7 dias	1,91	1,10	3,31	0,021		2,91	1,22	6,91	0,016
≥ 8 dias	1					1			
Usou PEP ou teste anti-HIV previamente									
Não	1,49	0,92	2,41	0,110		1,89	0,99	3,60	0,052
Sim	1					1			
Uso preservativo na primeira relação									
Sim	1,83	1,15	2,90	0,010		1,27	0,69	2,35	0,450
Não	1					1			
Autoavaliação do risco ^c^									
≥ 7 (alto)	2,02	1,18	3,44	0,010		0,42	0,17	1,03	0,059
1 a 6 (baixo)	1					1			
Conhecia PrEP									
Sim	2,01	1,10	3,91	0,023		1,14	0,56	2,32	0,726
Não	1					1			

OC: organização comunitária; IDH: Índice de Desenvolvimento Humano; CTA: centro de testagem e aconselhamento; PrEP: profilaxia pré-exposição sexual; PEP: profilaxia pós-exposição sexual.

a Índice de Desenvolvimento Humanos por Unidade de Desenvolvimento Humano, para residentes na cidade de São Paulo; e IDH municipal para residentes em outros municípios.

b Tempo ente primeiro contato com criação de demanda e prescrição de PrEP.

c Pergunta: Como você avalia seu risco de se infectar com o HIV, sendo 1 o risco mais baixo e 10 o mais alto?

Nenhuma das características relacionadas às práticas sexuais e a estar em contextos de maior vulnerabilidade para a prevenção (prostituição e uso de drogas) contribuiu para diferenciar os adolescentes das OC e CTA-centro.

A cor/raça não se mostrou associada a adolescentes das OC. Porém, ao ser incluída no modelo (dados não apresentados), essa variável alterou associações com o IDH, idade e autoavaliação do risco. Essas alterações foram mais expressivas na OC-centro, com a perda da associação com o IDH (p = 0,071) e aumento da chance de iniciar PrEP entre 15 e 17 anos (p = 0,044). Na OC-periferia, a associação ocorreu com a menor autoavaliação de alto risco (p = 0,049).

## DISCUSSÃO

Mostramos que a oferta de PrEP em organização da sociedade civil promoveu um acesso mais oportuno aos adolescentes socialmente vulnerabilizados. Particularidades dos locais e das organizações foram importantes para favorecer, na periferia, numa OC que trabalhava com a população geral, a inclusão de adolescentes mais jovens, residentes na comunidade e com menor acesso à prevenção; e, no centro, numa instituição LGBTQIAPN+, adolescentes mais informados, com maior percepção do risco e provenientes de diferentes locais da cidade. A oferta em um contexto comunitário, porém, não propiciou a inclusão de uma maior proporção de adolescentes com práticas sexuais de maior risco para o HIV.

Diferentes formas de ofertar PrEP próximo à comunidade têm mostrado efeitos positivos para pessoas LGBTQIAPN+, como as realizadas em farmácias, por pares ou na atenção básica ^4^^,^^5^^,^^23^ . Optamos por um modelo que valorizou uma oferta legitimada por organizações resultantes da ação de cidadãos, envolvendo pares e prescrição por enfermagem. Esse tipo de oferta tem sido relacionado à maior capacidade de atingir populações afetadas pelo HIV, incluindo adolescentes; aumentar taxas de iniciação da profilaxia e melhorar o contínuo de PrEP, em contextos diversos ^8^^,^^10^^,^^23^ . Esses resultados são atribuídos na literatura à maior proximidade com os valores e necessidades da população, conveniência de acesso e adoção de protocolos mais específicos e próximos a um cuidado integral, resultando numa maior capacidade de lidar com barreiras estruturais, como a cisheteronormatividade, o estigma, a violência e/ou o despreparo organizacional e profissional ^6^^,^^24^^,^^25^ . Esses fatores têm dificultado, em especial nos serviços de saúde mais convencionais, a construção de um ambiente mais acolhedor à prescrição de PrEP, comprometendo, entre outros aspectos, a revelação da orientação sexual e a manifestação da intenção de uso de métodos preventivos ^6^^,^^10^ .

Ademais, o protocolo de PrEP adotado no presente estudo centrou na enfermagem e nos educadores-pares todo o processo do cuidado, contrastando com o serviço convencional, no qual a prescrição da profilaxia envolveu diferentes etapas e somente profissionais de saúde. É possível que isso tenha favorecido uma oferta mais personalizada e amigável. Nesse sentido, estudos têm evidenciado que a enfermagem possui alta aceitação em PrEP, devido a um menor julgamento e maior proximidade com os indivíduos ^11^^-^^13^ . O mesmo é destacado para educadores-pares ^6^^,^^23^ , na medida em que reduzem o constrangimento de jovens, reforçam o protagonismo e facilitam o compartilhamento de experiências sobre sexualidade, prevenção e cuidado.

Um perfil particular de acesso e de características dos adolescentes também foi observado para cada OC. Essas diferenças provavelmente refletiram especificidades do local e do tipo de atuação de cada instituição. A inclusão de adolescentes mais jovens e com menor acesso à prevenção na OC-periferia pode ter sido favorecida pelo perfil da população local ^19^ e pelo tipo de atuação da OC voltado à população geral. Esse último aspecto pode ter reduzido o receio dos adolescentes de sofrerem com o estigma associado ao HIV e à comunidade LGBTQUIAPN+. Em Vancouver e Londres ^6^^,^^25^ , por exemplo, o receio do estigma foi uma das principais razões para HSH não procurarem um serviço na comunidade ou próximo da área de residência. De forma análoga, a inclusão de adolescentes mais preocupados com o HIV na OC-centro pode ter sido favorecida pelo fato de ser uma organização LGBTQUIAPN+, que tende a reunir pessoas mais engajadas e conscientes da temática. Além disso, a região central da cidade é historicamente reconhecida como menos hostil às expressões de sexualidades e gêneros não hegemônicas ^18^ , sendo um polo de atração para aquele que buscam ambientes mais próprios para a cultura LGBTQUIAPN+.

Esses resultados convergem com a concepção de que serviços com diferentes características podem fortalecer a universalização e equidade em PrEP ^4^^,^^5^ , especialmente em regiões com menor IDH ^1^^,^^26^ .

A ausência de diferenças no perfil de vulnerabilidade ao HIV entre instituições analisadas contrasta com outras investigações que identificaram maior frequência de risco nas estratégias comunitárias ^10^^,^^27^ . Embora haja diferenças entre os serviços, é possível que as estratégias de criação de demanda extramuros ^20^ , desenvolvidas nas três instituições, tenham contribuído para tornar o perfil das clientelas mais homogêneo. Outro aspecto a considerar é que a identificação de algumas diferenças pode ter sido dificultada pelo tamanho da amostra. Reforça-se, por último, o fato de os adolescentes incluídos no estudo terem uma prevalência de risco discretamente mais elevada do que a média dos usuários de PrEP de serviços da rede pública brasileira ^28^ .

Diferenças também não foram observadas para a inclusão de aTTrans, apesar da proporção dessa população ter sido cerca de 40% maior na OC-periferia, do que nos demais serviços. Atribuímos esse maior número à intensificação de estratégias voltadas a essa população na OC-periferia, como grupos de discussão e ações afirmativas de gênero. As barreiras de acesso para aTTrans, entretanto, são múltiplas e interconectadas, se estendendo do receio da testagem de HIV aos aspectos estruturais ^29^ , limitando o alcance de ações que abordam parcialmente a problemática.

Nessa mesma direção, destacamos a cor/raça que, embora não associada aos adolescentes das OC, influenciou a associação com outros importantes quesitos relacionados ao acesso, como IDH, percepção do risco e idade de início de PrEP. Isso reforça a necessidade de integrar nas estratégias preventivas a diversidade de cor/raça existente no Brasil (ex: integrar pessoas negras nas equipes de saúde e priorizar essa comunidade nas intervenções), dando respostas ao racismo estrutural.

Os resultados do presente estudo devem ser interpretados considerando seus limites. Fatores contextuais não controlados, como segurança, transporte ou violência relacionada ao gênero e/ou orientação sexual podem influir diretamente no acesso e definir as características da clientela de uma instituição. Há de se considerar, entretanto, que os resultados foram observados a partir de OC com características substancialmente distintas; e que as atividades chaves para favorecer o acesso foram, igualmente, desenvolvidas na comunidade e no serviço convencional, como: criação de demanda, treinamento dos profissionais; e apoio financeiro para transporte. Adicionalmente, incluímos na análise variáveis que refletem o contexto de forma mais abrangente, como IDH e distância moradia-serviço.

Em conclusão, a oferta comunitária, ao se aproximar do território e de organizações da sociedade civil, pode ser estratégica às políticas de prevenção, especialmente no momento de incorporação de novas tecnologias. Isso pode favorecer a ampliação de um acesso equitativo à PrEP para adolescentes que se encontram no início da trajetória sexual e da aprendizagem do autocuidado.
